# Legislators’ roll-call voting behavior increasingly corresponds to intervals in the political spectrum

**DOI:** 10.1038/s41598-020-74175-w

**Published:** 2020-10-15

**Authors:** David Schoch, Ulrik Brandes

**Affiliations:** 1 The Mitchell Centre for Social Network Analysis, The University of Manchester, Manchester, M13 9PL UK; 2 Social Networks Lab, Department of Humanities, Social and Political Sciences, ETH Zürich, Zürich, Switzerland

**Keywords:** Computational science, Complex networks

## Abstract

Scaling techniques such as the well known NOMINATE position political actors in a low dimensional space to represent the similarity or dissimilarity of their political orientation based on roll-call voting patterns. Starting from the same kind of data we propose an alternative, discrete, representation that replaces positions (points and distances) with niches (boxes and overlap). In the one-dimensional case, this corresponds to replacing the left-to-right ordering of points on the real line with an interval order. As it turns out, this seemingly simplistic one-dimensional model is sufficient to represent the similarity of roll-call votes by U.S. senators in recent years. In a historic context, however, low dimensionality represents the exception which stands in contrast to what is suggested by scaling techniques.

## Introduction

Numerous scaling techniques for roll-call votes have been developed over the last decades^1–5^, with NOMINATE arguably being the model example^6,7^. The approaches can differ profoundly in their technicalities, but ultimately, they all pursue the same objective. Produce an embedding of legislators in a low dimensional space which estimates their *ideal points*, reflecting, in a way, their ideological alignment. The ideal points are commonly derived from observed voting behavior. The substantive assumption is that legislators who consistently co-vote, i.e. they cast the same vote, have comparable political preferences and thus should be placed more closely together than those that have more dissimilar voting patterns.

Sooner or later, any scaling technique is faced with the questions: “How many dimensions are needed for a faithful embedding?” and “what do the dimensions actually mean?”. Poole and Rosenthal, the creators of NOMINATE, illustrate that in spite of all the complexities in politics, roll-call voting in U.S. Congress can be explained by no more than two dimensions throughout most of the American history, with a recent collapse into one single dimension. The first (or only) dimension is interpreted as the “left-right” or “liberal-conservative” spectrum of political ideology while the second picks up differences within political parties over historical issues such as slavery or civil rights.

While the primary purpose of scaling techniques is to produce a comprehensive map of political positions, they also allow to study the phenomenon of polarization. The growing distance between the means of parties in the first dimension of these maps illustrates the expanding ideological divides in the U.S. Congress, stimulating the discussion that the two major U.S. parties, the Democratic and Republican party, have become more deeply divided on ideology and governance issues over the past decades^8–14^.

Amid the literature on polarization of the U.S. Congress also exists a large body of research that examines the phenomenon from a network perspective^15–25^. Network ties among legislators are derived from co-voting behavior, bill co-sponsorships or joined press events and, predominantly, clustering techniques are employed to uncover cohesive groups of like-minded legislators. Independent of the underlying data, a consistent finding is that cohesive groups within the network approach party affiliation over time, providing further evidence that the U.S. Congress is becoming increasingly polarized and bipartisan consensus seems to disappear.

The work presented here combines the network-analytic approach with the technical idea of scaling techniques to represent members of U.S. Congress in a simplified structure. The main difference to previous work is that we use intervals and their overlap rather than points and their differences to conceptualize ideological positions. We develop the concept of *political niches* based on known results from intersection-graph theory, particularly from studies on interval graphs, applied to networks that connect legislators with overlapping political views. These networks are used to assess the dimensionality of the underlying structure, derive ideological alignments of legislators, and evaluate polarization within the Senate from the 81st to the 116th Session (up to May 2020) based on roll-call voting records.

## Notation and terminology

We consider undirected networks $G=(V,E)$ with vertex set *V* and edge set *E*. The cardinalities are denoted by $n=|V|$ and $m=|E |$. A *maximal clique* of a network $G=(V,E)$ is a fully connected subgraph that cannot be extended by including any other vertex. The *clique-membership matrix* is a $k \times n$ matrix *M* where *k* is the number of maximal cliques and *n* is the number of nodes. An entry $M_{ij}$ is one if and only if the *i*th clique contains node *j* and zero otherwise.

The *Laplacian matrix* *L* of a non-negative matrix *A* is defined as $L=D-A$, where *D* is a diagonal matrix with entries $D_{ii}=\sum _j A_{ij}.$ The minimum positive eigenvalue of a Laplacian matrix with an eigenvector orthogonal to the vector of all ones is called the *Fiedler value*. The corresponding eigenvector is referred to as the *Fiedler vector*.

A (0, 1)-matrix *A* has the *consecutive one’s property* (C1P) if there exists a permutation of the rows such that the 1s in each column occur consecutively.

## The concept of political niches

In this section, we introduce the concept of political niches and illustrate how they can be reconstructed based on networks inferred from roll-call voting patterns.

### Political niches and overlap networks

A common feature of scaling techniques is that they conceptualize ideologies as points in a space that is partitioned into two regions by the yea- and nay-votes on each bill. The lower the dimensionality of that representation, the less accurately the partitions will match empirical voting. An alternative representation proposed here starts from pairs of legislators with overlapping voting behavior and represents them in a multidimensional structure in which coordinates are replaced by tolerance intervals. Ideal points thus become axis-parallel boxes, which we refer to as the *political niche* of legislators. This notion is derived from an ecological context, where the living environment of species is described as a box in a *d*-dimensional space spanned by ecological factors^26^. Similar concepts have also been used in the context of social organizations to describe the socio-demographic niche of their members^27^.

**Figure 1:**
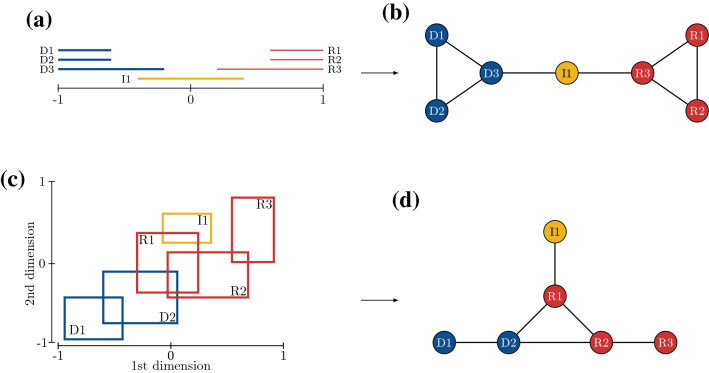
Hypothetical niche representations of legislators. Top row shows a one-dimensional example with (**a**) the respective interval representations of political niches and (**b**) the niche overlap network. Bottom row illustrates a two dimensional case with (**c**) the political niches as boxes and (**d**) the niche overlap network.

In the simple one-dimensional case, political niches are represented by a single interval on the real line. An example is shown in Fig. 1a. A two-dimensional example is given in Fig. 1c. Independent of the dimensionality, political niches may overlap for legislators with compatible political views. These overlaps can be used to define the so called *niche overlap networks* (cf. Fig. 1b,d), where legislators are connected by a tie if their niches overlap. In the case of a single dimensions, these networks are also known as *interval graphs*^28^ and, together with the higher dimensional cases, are part of a broader class of *intersection graphs*^29^.

We emphasize that ties in niche overlap networks have a substantively different meaning from ties in most other types of networks. Ties are often conceptualized with a positive connotation. The accumulation of ties is therefore considered beneficial, and forming new connections is better than not to^30^. Negative ties on the other hand suggest a more antagonistic relation among actors. Ties in niche overlap networks are neither positive nor negative, but rather express a form of equivalence, or similarity. In our case, they signify ideological agreement. Relations of this kind are sometimes referred to as *leveling ties*^31^.

### Constructing niche overlap networks from roll-call votes

Niche-overlap networks naturally arise from niche representations. In reality, of course, the political niches are latent idealizations and we cannot determine their overlap networks. In fact, the niches are what we wish to infer, if possible, from otherwise constructed overlap networks. A first step towards obtaining niche representations thus is the construction of such networks using appropriate proxy data which allows to uncover potential ideological overlaps among legislators. In accordance with the literature of ideal point estimates, we use the roll-call voting behavior to determine the networks based on the following assumption. If two legislators repeatedly cast the same vote, independent of approval or rejection, then there exist some commonalities in their underlying political ideologies. Thus, their niches overlap. If they consistently vote in opposing directions it is more likely that their political views are not compatible and their niches are distinct. This idea is implemented with the *stochastic degree sequence model* (SDSM)^32^. The model allows to reduce a weighted network, such as the co-voting network of legislators, to a binary one. The resulting binarized networks from the SDSM only include a tie between legislators if the number of co-votes is higher than expected by the null model, i.e. there is “significant co-voting” and hence compelling evidence that the underlying latent niches indeed overlap.

### Inferring political niches from overlap networks

Once the overlap networks are constructed from the roll-call voting records, we can proceed to construct the underlying political niches. The first question to ask is, how many dimensions are necessary to represent the networks as overlapping boxes. This question relates to the concept of *boxicity*, which is defined as the minimum number of dimensions necessary to represent it as an intersection graph of axis-parallel boxes^33^. Interval graphs are boxicity 1 graphs since they can be represented as overlapping intervals in one dimension. Deciding if a network is an interval graph can be done in linear time^34,35^. Practically, however, a method based on the fact that the clique-membership matrix of an interval graph has the C1P^36^ may be preferred. The permutation matrix $\Pi$ which induces the C1P ordering can be obtained by ordering the Fiedler vector of the Laplacian matrix of $MM^T$^37^. Hence, if the Fiedler vector of $MM^T$ induces a C1P ordering of *M*, then the network is guaranteed to be an interval graph, having boxicity 1. The interval representation is given by the locations of ones in each column in the reordered matrix $\Pi M$ (Further technical details and an example are provided in the Supplementary Information).

Networks of boxicity 1 are easy to identify and the niche representations are straightforward to compute. Deciding if a network has boxicity at most 2, however, is already NP-complete^38^ and methods to determine niche representations for boxicity 2 are rather involved^39^. Therefore, in general, we cannot hope to determine the most parsimonious representations of niche overlap networks if one dimension does not suffice. We resort to approximate solutions based on the structural divergence of a network from an interval graph. The more a network deviates from an interval graph, here measured in terms of violations of the C1P in the clique-membership matrix, the more dimensions are assumed to be necessary to describe the political niches of legislators. (Additional measures are discussed in the Supplementary Information.) Since the matrix does not have a C1P ordering for higher dimensional cases, there exists at least one column where a zero is surrounded by two ones. Such a “hole” is called a *Lazarus event*. The minimal number of Lazarus events induced by any permutation is referred to as the *Lazarus count*^40^. Finding the optimal permutation is computationally intractable but upper bounds can be obtained, for instance, with local search methods such as simulated annealing. To make results comparable across networks, we normalize the raw Lazarus counts by the number of columns (i.e., nodes) of the clique-membership matrix.

### Niche point projections

If the boxicity of a niche-overlap network is higher than the dimensionality of its representation, we can try to obtain a representation with a small number of misrepresented overlaps, for example using random super graphs^41^. A different and computationally feasible alternative brings us back to fixed point representations, albeit based on the overlap networks.

Let *A* be the $n\times n$ adjacency matrix of a niche overlap network, where $A_{ij}=1$ if the niches of legislator *i* and *j* overlap. We seek a representation $x\in \mathbb {R}^n$ of niches that yields an approximation of their position in the first dimension. Overlapping niches of legislators *i* and *j* should have similar scalar representations $x_i$ and $x_j$ while non-overlapping niches should be placed at a distance. This can be formalized with the constrained minimization problem

1 $$\begin{aligned} \min \limits _{x} \sum _{ij} A_{ij} (x_i-x_j)^2 \quad s.t. \; \sum _i x_i=0 \text { and } \sum _i x_i^2=1. \end{aligned}$$

The problem can be written in terms of the Laplacian matrix *L* of *A* as $x^TLx$ and a solution is given by a Fiedler vector. Although we cannot recover the true extend of niches, we can thus still evaluate how legislators are ordered along the principal left-right dimension using the Fiedler vector of *L*. To obtain a projection of the second dimension, we need to choose a vector *y* which minimizes the optimization problem in Equation 1 under the condition that *y* is normalized and orthogonal to *x*. A solution for this problem is given by the eigenvector associated with the second smallest non-zero eigenvalue of the Laplacian matrix. Further projections of higher dimensions can be computed analogously.

Note that these fixed points are conceptually different to those derived from traditional scaling techniques. They represent projections of niches into fixed points rather than actual ideal point estimates. The benefit of the niche point projections is that they allow us to define distances between niches. This enables us to assess polarization in the same way as scaling techniques by comparing distances between party means over time.

## Results

**Figure 2:**
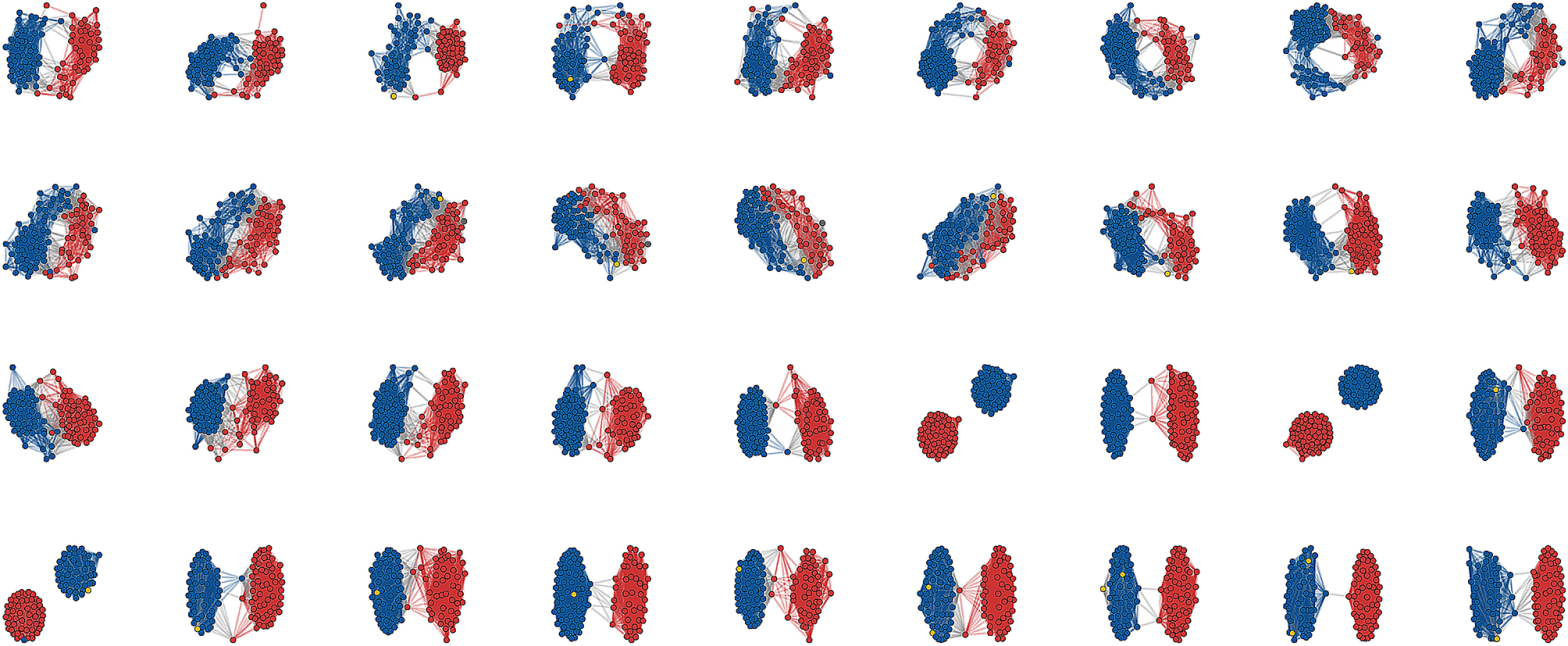
Niche overlap networks of senators inferred from roll-call votes with the SDSM for the 81st (top-left) to 116th Senate (bottom-right).

In this section, we examine the 81st to 116th Session (up to May 2020) of the U.S. Senate with the presented methodology. The niche overlap networks derived from roll-call voting records using the SDSM are shown in Fig. 2.

### Dimensionality

**Figure 3:**
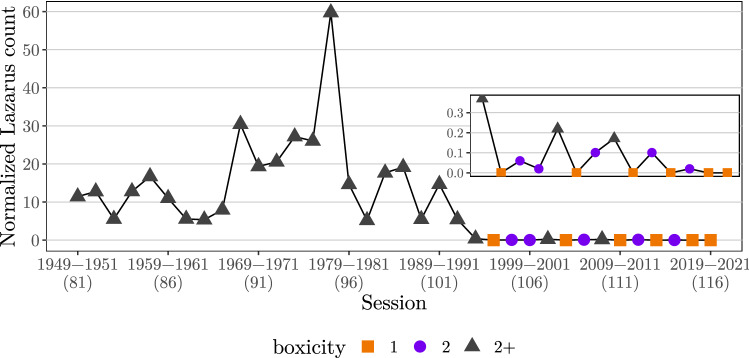
Upper bound for the normalized Lazarus counts obtained via simulated annealing of the niche overlap networks shown in Fig. 2. Point shapes and color indicate boxicity. Inset shows the 103rd–116th Senate in more detail.

Figure 3 shows the normalized Lazarus count for the niche overlap networks. Evidently, a majority of the networks are far from being low-dimensional. The high Lazarus counts suggest that, in most cases, at least two dimensions are necessary to characterize the political niches of senators. However, we also observe a sharp decrease of dimensionality coinciding with the presidency of Ronald Reagan, which has been associated with an increase of polarization in both chambers of Congress^42^. Since the 104th Senate, six networks are indeed interval graphs, implying that a single dimension suffices to describe the political niches of senators. Of the remaining seven networks in this time period, we found that five have boxicity 2, i.e., niches can be represented as rectangles.

### Niche representation and equivalence classes

**Figure 4:**
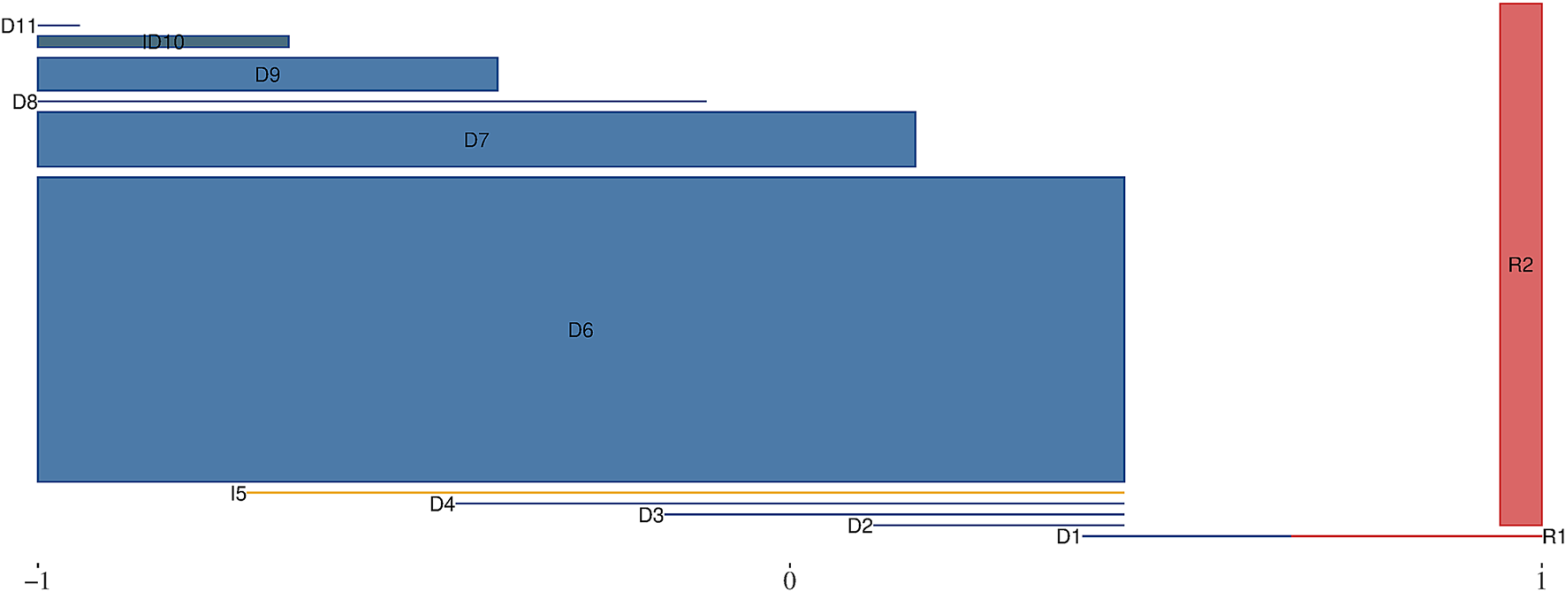
Interval representation of political niches for the 115th Senate. Senators with coinciding niches are merged together into rectangles. The height of rectangles only represents the size of the composite classes. Niches of interest are (R1) SM Collins (ME), (D1) J Manchin (WV) and (D2) MK Heitkamp (ND). (A fully annotated figure can be found in the Supplementary Information.).

Figure 4 shows the political niches for the 115th Senate, where senators with coinciding niches are merged together. (The remaining one and two dimensional representations can be found in the Supplementary Information). Evidently, and in line with scaling techniques, the dimension can be characterize as the left-right or liberal-conservative spectrum. The intervals itself reveal some significant differences between Democrats and Republicans. All but one Republican senator reside on the right end and are characterized by coinciding intervals. This agreement is the consequence of a property of the underlying niche overlap network. If two senators are connected with each other and to exactly the same set of other legislators, then they are *strongly structurally equivalent*. This implies that they are not distinguishable by the network structure alone and they occupy exactly the same political niche. The large block of equivalent Republicans may indicate that intra-party guidelines overrule most of the political attitudes of individual senators. The exception is Sen. Collins, who has been described as a “maverick” Republican by the media, voting against her party at various occasions^43^. Democrats and Independents can be grouped into eleven blocks of increasingly bipartisan senators. Intervals that stand out are those of Sen. Heitkamp and Sen. Manchin. Both have previously been described as being more centrist or even conservative democrats^44,45^.

**Figure 5:**
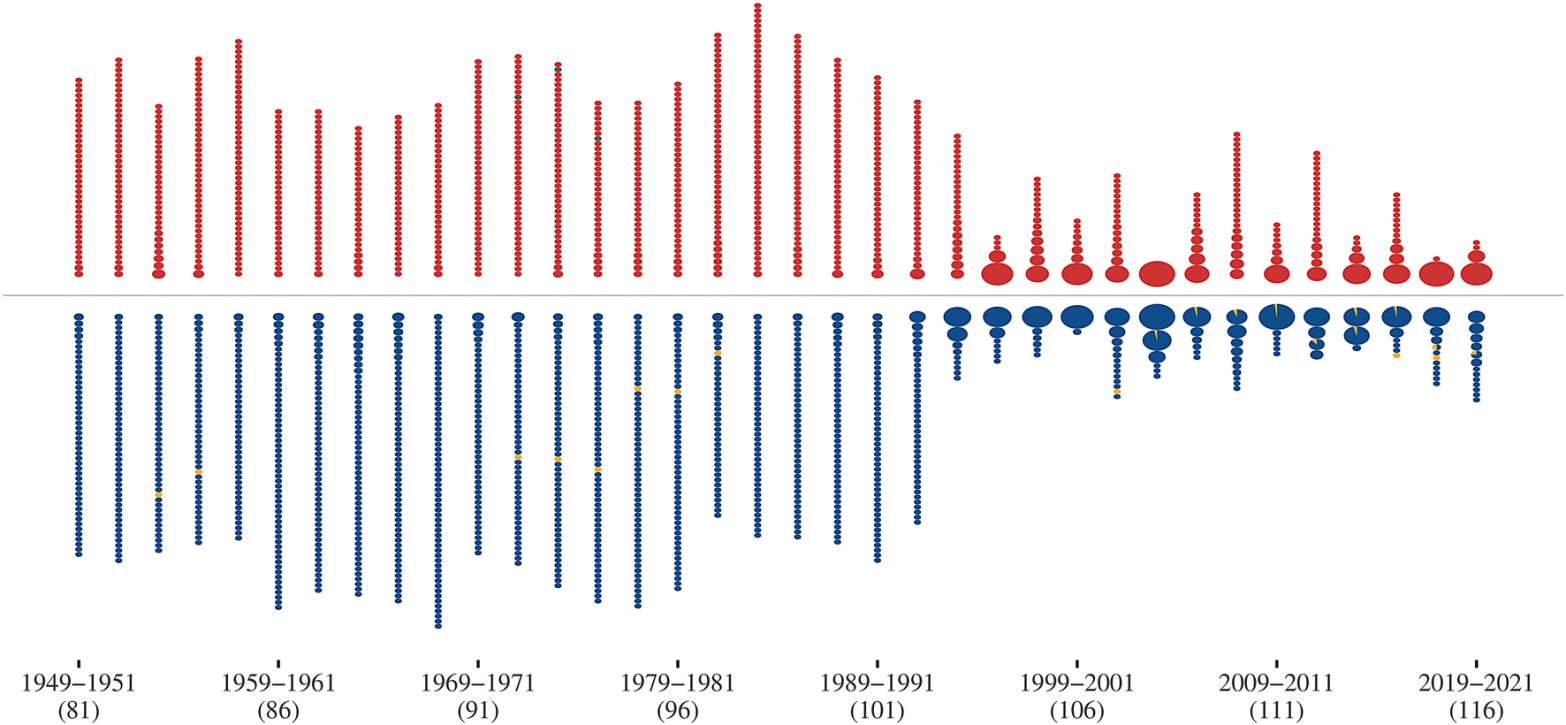
Classes of strongly structurally equivalent senators over time. Each pie chart represents an equivalence class and its composition. The radius is proportional to the size of the equivalence class.

The discrepancies in structural equivalent legislators between the parties is not only visible in the 115th Senate. Figure 5 shows the compositions and sizes of all equivalence classes over time. First, note that the sharp decrease in dimensionality shown in Fig. 3 coincides with a sharp increase in the size of equivalence classes for both parties. This change in the composition of equivalent blocks in fact offers a partial explanation for the sharp decrease in dimensionality of the networks. Groups of equivalent legislators occurred very infrequently before the 102nd Senate. Most senators form singleton classes, i.e. their niches are unique. After the 102nd session, large blocks of legislators with equivalent niches start to form. This may be an indicator that the sincere voting assumption, i.e. voting for the outcome closest to their ideal point^7^, is no longer valid and legislators vote according to intra-party guidelines, hence collapsing other dimensions into the left-right spectrum.

While the number of equivalence classes decreases for both parties, it does so with different intensity. Republicans remained more diverse, i.e. there are more classes which are smaller in size, with less indication of intra-party guidelines affecting sincere voting. This changed with the Trump administration, where Republicans dropped from 13 classes in the 113th Senate into two and, so far, four groups in the 115th and 116th Senate, respectively. Democrats, on the other hand, increased from 5 large classes to 11 and 14 smaller groups of equivalent legislators.

### Polarization

**Figure 6:**
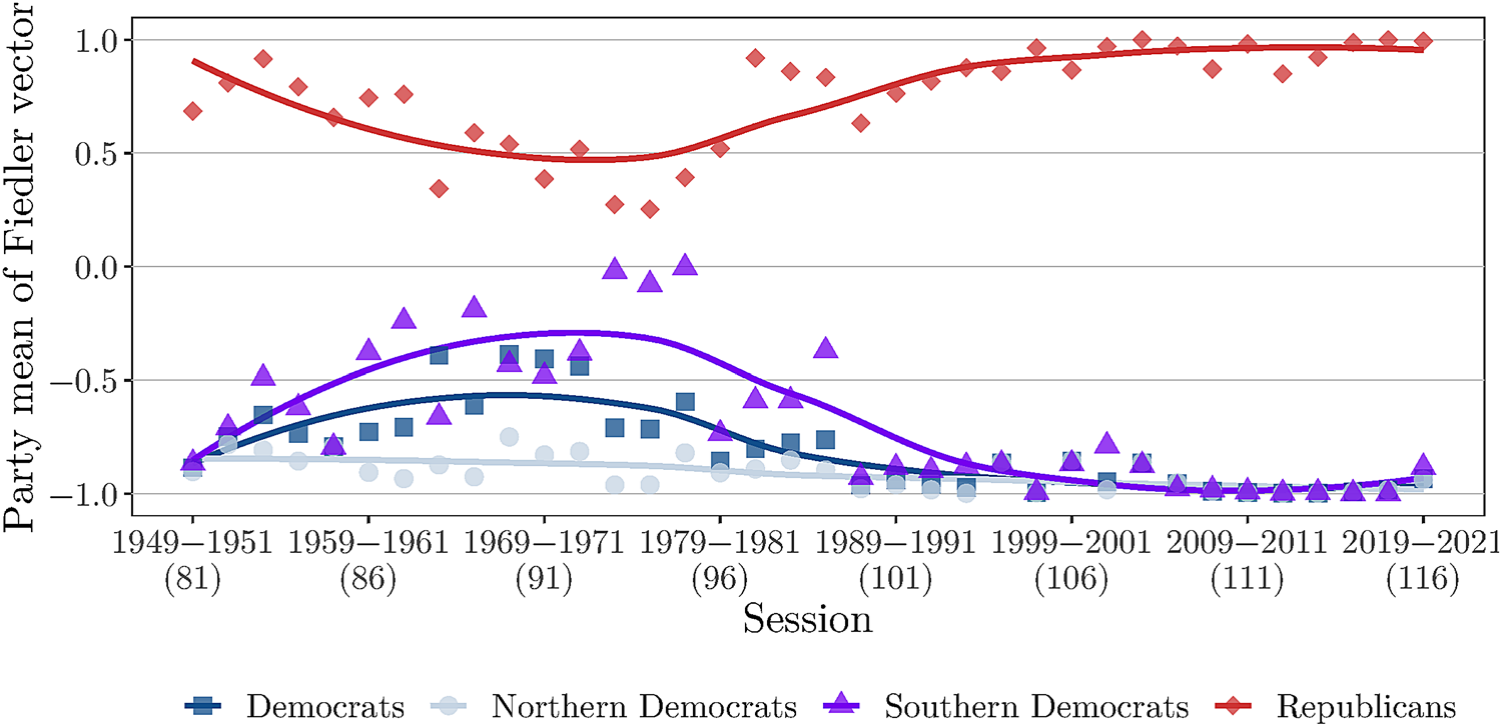
Mean of the Fiedler vector for Democrats (all, Northern and Southern), and Republicans.

To quantify the growing polarization over time, we employ the one dimensional niche point projections obtained from the Fiedler vector. Figure 6 shows the mean of the Fiedler vector for the two parties over time. Evidently, there is a clear shift of parties toward the respective extreme ends of the spectrum. The result confirms what has been reported before, i.e. the Senate has become considerably more polarized in recent years. The respective distance is consistently higher than 1.5 since the 104th Congress and thus close to the theoretical maximum of 2. A division of the Democratic party into Northern and Southern Democrats (The “South” being the 11 states of the former Confederacy together with Kentucky and Oklahoma^46^), reveals a partial explanation for the increasing polarization. While Northern Democrats have been liberal throughout the considered time period, their Southern counterpart maintained a fairly conservative stance till the mid 1980s^47^. The emergence of a competitive Republican party and greater involvement of black voters, however, changed the political landscape in the south and Democrats began to liberalize their voting record^48^.

## Discussion

In this study, we presented a set of methods that allow the examination of political ideologies based on network-analytic concepts. While our approach does share some similarities with more established scaling techniques, it differs in three key aspects.

Firstly, dimensions are constructed only after approximating co-voting behavior in an overlap network. Although computationally intractable for higher values, the number of dimensions necessary for exact representation can be determined in principle. In contrast, scaling techniques seek to approximate distances between ideal positions in few dimensions. The quality of representation is determined either by comparative model fitting using BIC, AIC or the aggregate proportional reduction in error, or using heuristics such as the “elbow-test” or scree plots. Regardless of how the dimensionality is determined, it is mostly up to the researcher to choose the set of dimensions that approximate the data best. Remaining errors or variance are commonly attributed to noise. Our approach, on the other hand, requires to find the optimal number of dimensions that represents all the data without dropping any information.

Secondly, ideological positions are not conceptualized as points in a political space build to represent distances, but as boxes in a discrete structure representing overlap. Instead of (weak) orders of points in each dimension we obtain (weak) interval orders. Since interval orders are more general they may require fewer dimensions, but it will be an interesting aspect of future work to determine to which degree this plays out empirically.

Thirdly, scaling techniques are derived from the partitioning property of roll-call votes, and thus do not generalize easily to other elements of political action. Niche-overlap networks, on the other hand, can be constructed from other forms of legislative data that reveals shared political positions. Bill co-sponsorships, for instance, pose one viable alternative. Data on co-sponsorship is far more comprehensive than roll-call votes, since only a small fraction of proposed bills are actually voted on. It is therefore not surprising that especially network-analytic studies predominantly rely on co-sponsorship^24^. In the present study, we choose roll-call votes to remain comparable with traditional scaling techniques. (The Supplementary Information includes the results for bill co-sponsorship data.).

A limitation of our approach that should be addressed in future work is the lack of a simultaneous representation of bills. This restricts the potential for predictive modeling, i.e., for forecasting roll-call voting behavior of senators. In contrast to ideal point estimates, we do not embed bills in the same space which does prevent the ability to make predictions. This is a natural limitation of using one-mode projections, where information on the other mode is lost. A potential solution is to employ the dual projection approach^49^, but the interpretation appears to be less straightforward than with scaling.

Observationally, our results confirm much of what has been obtained with ideal point estimates. Still, it is striking that senators can be represented in an interval order for most sessions of congress in recent years. Interval graphs are rarely encountered in empirical research unless there is an underlying mechanism that is prone to produce them. While conflict graphs are a natural model in many technical applications, known examples for humans and other animals are largely restricted to food webs of various ecosystems^26^. Even there, however, it is difficult to associate a substantive meaning with that dimension, i.e., to identify the environmental factor it represents. In our case, we are confident that it can be interpreted as the left-right political spectrum.

The assumption of a general tendency towards low dimensionality is, however, contested. Besides the six one-dimensional sessions, only another five Senates could be confirmed to have a dimensionality of two. The remaining networks may be far from having low dimensionality, implying that more than two dimensions are necessary to describe the niches of senators. This observation strengthens recent results which suggest that scaling techniques are not always able to capture the true underlying dimensionality and yield a false sense of regularity^50^.

The observed sharp drop in dimensionality over time could be explained in part by the increasing number of structurally equivalent legislators in the niche-overlap networks. That is, senators in both parties vote almost exactly the same and thus occupy the same political niche. Substantively, this indicates that the assumption of sincere voting, which most scaling techniques rest upon, is less likely to be valid. It suggests that partisanship is now the dominant factor underlying voting behavior of senators. Clearly, more substantive arguments are needed to support this claim, but the fact that an interval order is generally sufficient to represent co-casting of roll-call votes appears difficult to explain otherwise.

## Methods

The roll-call voting records for the 81st to 116th Session (up to May 2020) of the Senate were obtained from https://voteview.com^51^. R scripts to download the data and replicate our study are provided in the Supplementary Information. The document also contains additional technical details and mathematical justifications on low dimensional intersection graphs, specifically interval graphs.

### Stochastic degree sequence model

The SDSM is a model that allows to extract a *binary backbone* from one-mode projections of two-mode networks. The voting behavior of senators can be modeled as such a network. Denote by *B* the $n\times m$ voting matrix, where *n* is the number of legislators, *m* the number of bills, and the entries $B_{ik}$ represent the vote of legislator *i* on bill *k* (“yea”, “nay” or abstain). We use similar preprocessing steps as done by NOMINATE, i.e. we exclude all votes in our dataset where the minority is below 2.5%^6^. NOMINATE also removes all legislators from the analysis who voted on less than 20 bills. We opt for a more conservative threshold and require legislators to have voted on at least 50% of all roll-call votes.

From matrix *B*, we derive a two-mode network with $n+2m$ nodes, where the first mode consists of *n* legislators and the second contains two nodes per bill, indicating (dis)agreement. For instance, $B_{11}=1$ if legislator 1 voted yea on bill 1. If they voted nay, then $B_{11}=0$ and $B_{12}=1$. The adjacency is given by an $n \times 2m$ matrix *A*, where an entry $A_{ik}$ is one if legislator *i* is connected to the *k*th node of the second mode. The network is then projected to a weighted one-mode network (the co-voting network) of legislators with adjacency matrix $P=AA^T$, such that an entry $P_{ij}$ corresponds to the number of times legislator *i* and *j* casted the same vote. The SDSM is then used to binarize the matrix.

Initially, a binary outcome model is fitted which predicts the entries $A_{ik}$ based on the row and column sums of *A*. The choice of model ultimately depends on which model approximates the data best. In our case, the scobit model overall yields the best fit but differences to more traditional models such as the logit model are only marginal. The model coefficients are used to estimate the probabilities $p_{ik}$ of a legislator *i* connecting to a “Yea” or “Nay” vote on bill *k*. These probabilities can be used to construct random two-mode networks $A^*$, where $A^*_{ik}$ is the outcome of one Bernoulli trial with probability $p_{ik}$, and its projection $P^*=A^*A^{*T}$. To decide if a tie between *i* and *j* should be included in the niche overlap network, the SDSM model uses a Poisson Binomial distribution to estimates the probability for each $P^*_{ij}$ being equal to or above the observed value $P_{ij}$. The significance level was set to $\alpha =0.05$ in our analysis.

All computations involving the SDSM model were done with the R package *backbone*^52^. Additional robustness checks using different model parameters are provided in the Supplementary information.

## Supplementary information

Supplementary Information.
