# Supplementary material for: Legislators’ roll-call voting behavior increasingly corresponds to intervals in the political spectrum
Source: Sci Rep. 2020 Oct 15;10:17369. doi: 10.1038/s41598-020-74175-w (PMC7566643; doi:10.1038/s41598-020-74175-w)
Supplement: Supplementary file 1 — Supplementary Information. [file 41598_2020_74175_MOESM1_ESM.pdf]

Supplementary Information
Legislators' roll-call voting behavior increasingly corresponds to intervals in the political spectrum

David Schoch[1,*] **and Ulrik Brandes**[2]

[1]The Mitchell Centre for Social Network Analysis, The University of Manchester, Manchester, M13 9PL, United Kingdom
[2]Social Networks Lab, Department of Humanities, Social and Political Sciences, ETH Zürich, Switzerland
[*]david.schoch@manchester.ac.uk

This document includes additional materials for the article "Representing lawmakers' voting behavior as intervals in the political spectrum" by David Schoch and Ulrik Brandes

Contents

Data and Code 1
Intersection graphs 2
Interval graphs . . . 2
Boxicity 2 graphs . . . 3
Stochastic degree sequence model 4
Polytope model . . . 4
Minority filtering . . . 4
Alternative distance measures 5
Graph edit distance . . . 5
Run-length Lazarus count . . . 5
Niche representations 6
Interval representations . . . 6
Boxicity 2 representations . . . 10
Niche point projections . . . 15
Co-sponsorship networks 15
References 17

Data and R Code

All analyses were done in R[1]. Scripts to reproduce the results from the paper are provided on github (`http://github.com/schochastics/congress`).

Intersection graphs

This section includes some additional technical details on interval and boxicity 2 graphs. Proofs of stated theorems can be found in the original work.

Interval graphs

The following theorem provides the technical justification for using the clique-membership matrix to detect interval graphs.

Theorem 1 (Fulkerson & Gross[2]) *A graph $G = (V, E)$ is an interval graph if and only if the clique-membership matrix has the C1P.*

Note that interval graphs can be characterized in ways that do not involve the clique-membership matrix M. As stated in the main paper, we only employ Theorem 1 for practical reasons.

Definition 1 *An $n \times n$ matrix A is said to be a* Robinson matrix *if and only if it is symmetric and*

$$\begin{aligned} A_{ij} &\leq A_{ik} \qquad \text{for } j < k < i \\ A_{ij} &\geq A_{ik} \qquad \text{for } i < j < k. \end{aligned}$$

Note that the diagonal elements are not specified. If a matrix can be permuted into a Robinson matrix, then A is a *pre-Robinson matrix*.

The following two theorems yield the justification of using the Fiedler vector to identify interval graphs.

Theorem 2 (Atkins et al.[3]) *Let A be a pre-Robinson matrix with a simple Fiedler value and a Fiedler vector with no repeated values. Let Π_1 (respectively, Π_2) be the permutation matrices induced by sorting the values in the Fiedler vector in increasing (decreasing) order. Then $\Pi_1 A$ and $\Pi_2 A$ are Robinson matrices and no other permutations of A produce Robinson matrices.*

The theorem can be generalized to allow for repeated entries in the Fiedler vector[3].

Theorem 3 (Kendall[4]) *Suppose A is a $(0,1)$-matrix with the C1P. The permutations of the rows of A which produce consecutive one's correspond exactly to the permutations which, when applied simultaneously to rows and columns, put AA^T into Robinson form.*

Theorem 2 and 3 imply that the Fiedler vector of MM^T yields a C1P ordering for M if the associated network is an interval graph. Once M is permuted, the interval representation for each node can simply be inferred from the location of ones in the respective columns.

An illustration of the outlined steps to recover the interval representation is shown in Figure 1.

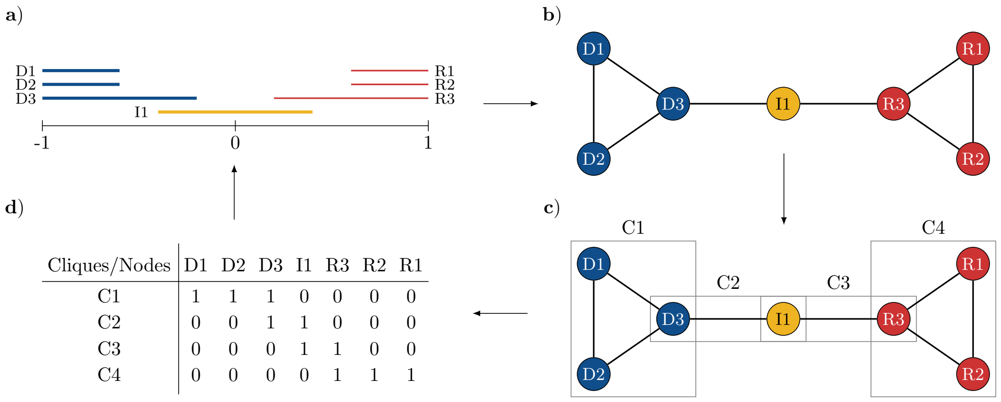

Cliques/Nodes	D1	D2	D3	I1	R3	R2	R1
C1	1	1	1	0	0	0	0
C2	0	0	1	1	0	0	0
C3	0	0	0	1	1	0	0
C4	0	0	0	0	1	1	1

Figure 1. Connection between a) interval representations, b) niche overlap network, c) maximal cliques and the d) clique-membership matrix.

Boxicity 2 graphs

The method used in the main paper to detect boxicity 2 graphs is based on the work by Quest and Wegner[5]. Let A be the $n \times n$ adjacency matrix of a niche overlap network $G = (V, E)$. For each $h \in \{1, \dots, n\}$, define the vertex sets

$$J_h = \{v_k \ : \ k = h \text{ or } (k > h \text{ and } A_{ik} = 1 \text{ for some } i \leq h)\}.$$

Each vertex h induces a new clique-membership matrix $M^{(h)}$ with entries

$$M_{jk}^{(h)} = \begin{cases} M_{jk} & v_k \in J_h \text{ and for some } v_r \in J_h \ : \ M_{jr} = 1 \text{ and } A_{kr} = 0 \\ * & else, \end{cases}$$

where "*" is a placeholder for entries in $M^{(h)}$ that are irrelevant. Asterisks are used instead of suppressing the those entries such that all matrices keep the same dimensions as the original clique-membership matrix M. The matrices $M^{(h)}$ are considered to be $(0,1)$-matrices due to the insignificance of the asterisks. A matrix $M^{(h)}$ has the C1P if there is no zeros between ones in any column, skipping all asterisks.

Theorem 4 (Quest and Wegner[5]) *A graph $G = (V, E)$ with adjacency matrix A and clique-membership matrix M is a boxicity 2 graph if and only if there exists a pair of permutation matrices Π_1 and Π_2 such that $\Pi_1 A$ and $\Pi_2 M$ induce clique-membership matrices $M^{(h)}$ with the C1P for each $h \in \{1, \dots, n\}$.*

The proof of sufficiency introduces a way to actually retrieve the box representation in two dimensional space. Relate the jth clique to the line $x = j$ and the sets J_h to the lines $y = n - h$. If $M_{jv} = 1$ and $v \in J_h$, we label the point $(j, n-h)$ with v. The box representation of v is then given by the convex hull of all points labeled v.

Although the theorem gives a characterization of boxicity 2 graphs, there is no way to determine the necessary permutations of A and M in an efficient way. In our analysis, we employ simulated annealing to determine permutation matrices Π_A and Π_M which yield an upper bound for the lazarus count of all clique-membership matrices $M^{(h)}$. If permutations are found that yield a Lazarus count of 0, then the graph is guaranteed to have boxicity 2. Note, however, that a nonzero count does not rule out the possibility of the graph having boxicity 2.

Stochastic degree sequence model

This section includes two robustness checks for the stochastic degree sequence model (SDMS), which was used to compute the niche overlap networks. Note that, in general, there is no satisfactory way to validate the quality of one-mode projections derived from different binarization techniques. In the absence of such a method, we use the Lazarus count of the resulting networks to compare the one-mode projections. The less it varies, the more stable are our results and thus independent from employed binarization methods.

Polytope model

The SDSM allows several different link functions to be used to fit a binary model on the roll-call data. In the main paper, the scobit model was used. Figure 2 shows the normalized Lazarus count for the networks computed with the "polytope" model, which is implemented in the R package *backbone*[6] and the preferred choice of the authors (personal communication). The figure shows that the Lazarus count does not vary notably by changing the model.

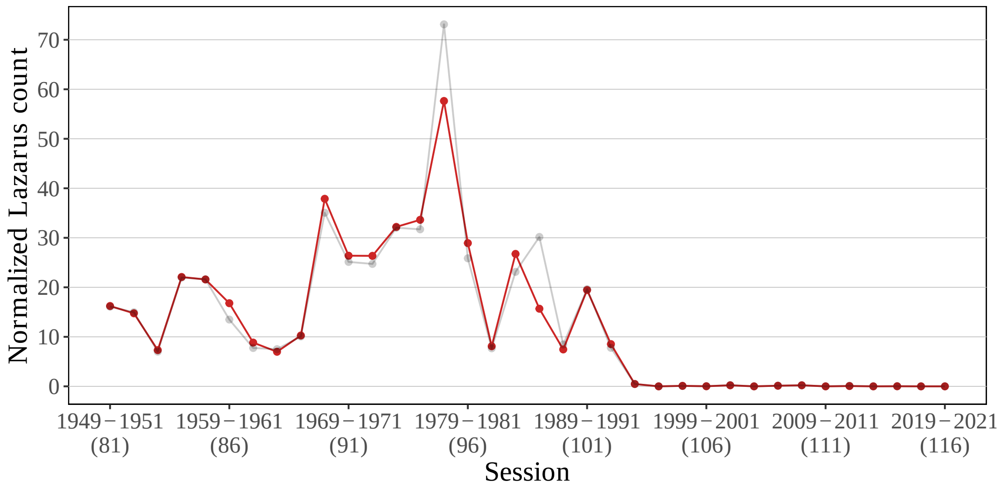

Figure 2. Upper bound for the normalized Lazarus counts obtained via the Fiedler vector for the niche overlap networks obtained using the polytope model (red) and the scobit model from the main paper (grey).

Minority filtering

In the main paper, we excluded all votes from the data where the minority is below 2.5%. This was done in order to be comparable with NOMINATE. From a technical perspective, however, the SDSM should be able to handle these cases without exclusion. Figure 3 shows the normalized Lazarus count for the networks computed without filtering in comparison to the filtered networks. Overall, the results do not vary significantly and no method clearly outperforms the other. We note though that in the unfiltered case, two senates are no longer one dimensional.

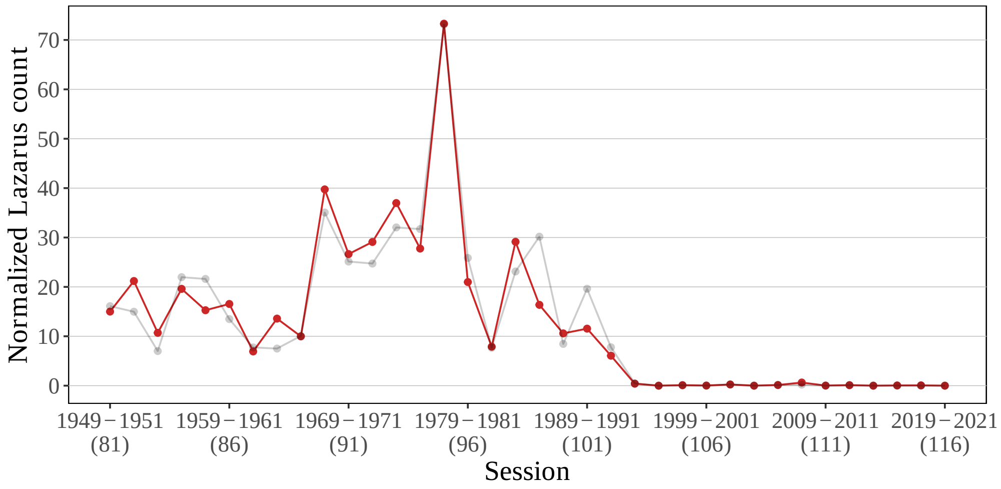

Figure 3. Upper bound for the normalized Lazarus counts obtained via the Fiedler vector for the niche overlap networks obtained from unfiltered roll-call votes (red) and the filtered networks from the main paper (grey).

Alternative distance measures to interval graphs

In the paper, we use the Lazarus count to assess the structural differences between a network and interval graphs. There are, however, others that could be employed which will be discussed in this section.

Graph edit distance

An alternative distance measure can be derived via graph edit distances, i.e. how many edges must be added/deleted in order to turn a graph into an interval graph. There exist at least three feasible instantiation of the interval edit problem: *interval graph completion* (only edge additions allowed), *interval graph deletion* (only edge deletions allowed), and *interval editing* (both edits allowed). All three, however, are NP-hard[7–9]. An approximation for the interval completion problem can be obtained via randomized interval supergraphs[10]. Given a graph $G = (V,E)$ and a permutation π of its vertices, we define a map $\mathscr{M}(G,\pi)$ which associates an interval supergraph $G_\pi = (V,E_\pi)$ to the pair (G,π) as follows. Let u be an arbitrary vertex and let $v \in N[u]$ be the vertex with $\pi(v) = \min_{w\in N[u]} \pi(w)$. Associate the interval $[\pi(v),\pi(u)]$ to the vertex u. It is easy to verify that the resulting graph G_π then is an interval supergraph of G.

This construction of interval supergraphs can be used to approximate the interval completion problem by solving the minimization problem

$$\min_{G_\pi \in \mathscr{M}(G,\pi)} |E_\pi \setminus E|, \tag{1}$$

using standard search heuristics such as simulated annealing. Figure 4 shows the lowest obtained edit distance for the non-interval niche overlap networks.

Run-length Lazarus count

The Lazarus count is not the only metric to assess "non-consecutiveness" of matrix columns. Recall that the Lazarus count is the sum of the total number of zeros between the first and last non-zero entry in each column. An alternative metric can be defined by summing up the number of consecutive series of zeros between the first and last non-zero entry in each column[11]. We refer to this metric as the *run-length Lazarus count* since it is equivalent to the Lazarus count of the run-length encoded columns. In many ways, the metric behaves similar to the Lazarus count. If the network is an interval graph, then the Fiedler vector of the clique-membership matrix induces the ordering such that the run-length Lazarus count is zero. An interesting aspect about the metric, though, is its connection to a specific traveling salesman problem. Let M be the $k \times n$ clique-membership matrix of a network $G = (V,E)$. Add two rows containing only zeros to M, one at the top and one at the bottom. Define the $k+2 \times k+2$ distance matrix D where entries D_{ij} correspond to the Hamming distance between the rows i and j of M. The solution of the travel salesman problem with distance matrix D gies the permutation which minimizes the run-length Lazarus count. For more technical details see[11].

Note that the run-length Lazarus count can be minimized efficiently given that exact solvers for the traveling salesman problem can easily handle problems of our size (i.e. instances with ≤ 1000 cities). Figure 5 shows the exact minimum run-length Lazarus counts, normalized by the number of nodes, which where determined with the Concorde TSP solver[12].

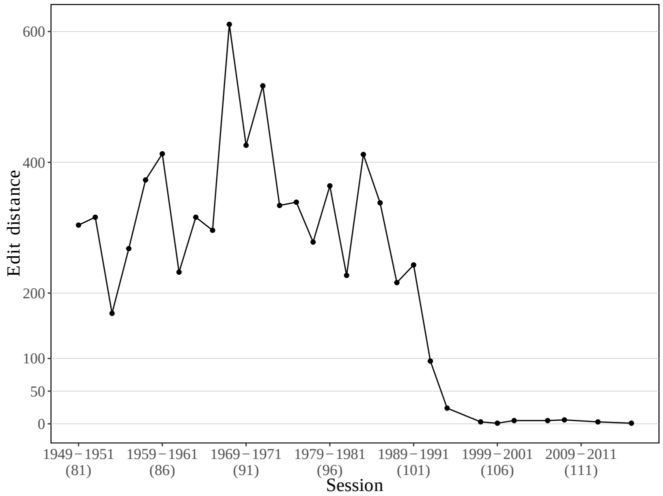

Figure 4. Upper bound for the interval completion instantiation for the niche overlap networks for the 81st-116th Senate. Known interval graphs are excluded.

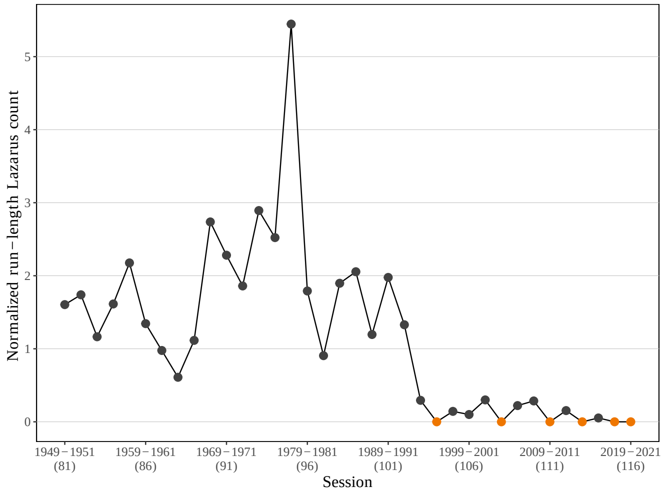

Figure 5. normalized run-length Lazarus counts of the niche overlap networks for the 81st-116th Senate. Orange points indicate that niches are intervals.

In general, there is no established rule on which metric to choose to assess the structural divergence of a graph from being an interval graph. The adequate choice may vary between applications. We opt for the Lazarus count since it appears to be more established in the literature but note that the presented alternatives would have given comparable results.

Niche representations

This section shows all niche representation of overlap networks that were found to be one or two dimensional, as well as the Fiedler vector representation used to assess polarization.

Interval representations

The figures below show the interval representations for the six niche overlap networks that were found to be interval graphs.

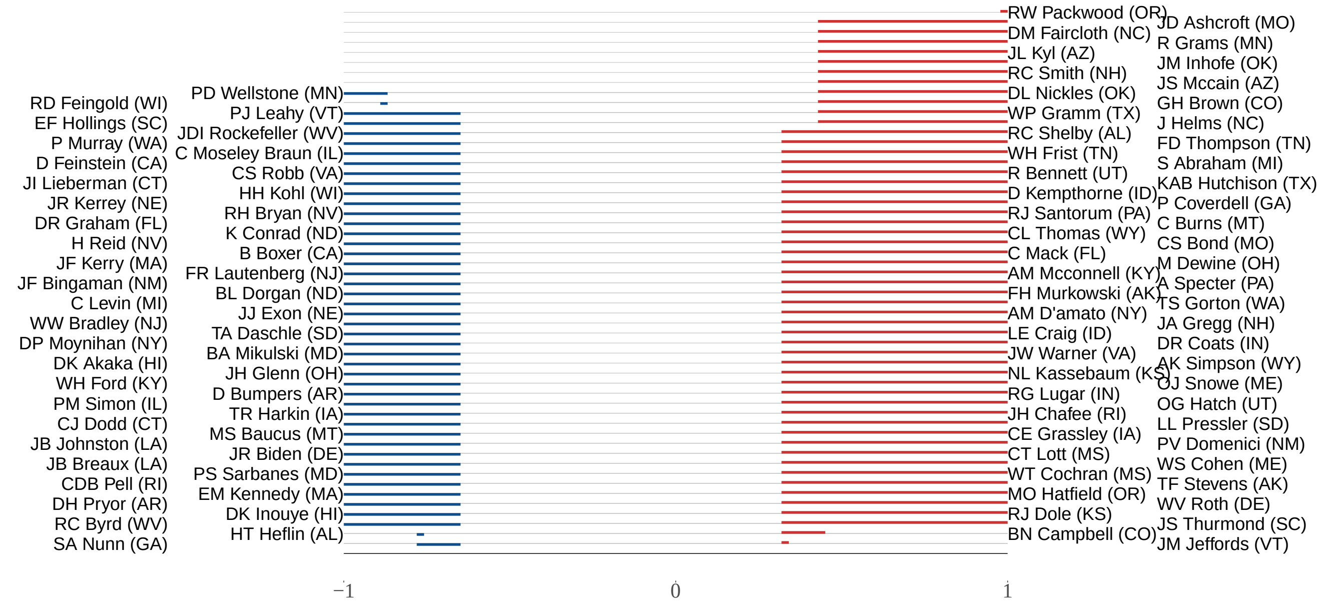

Figure 6. Interval representation of the 104th Senate.

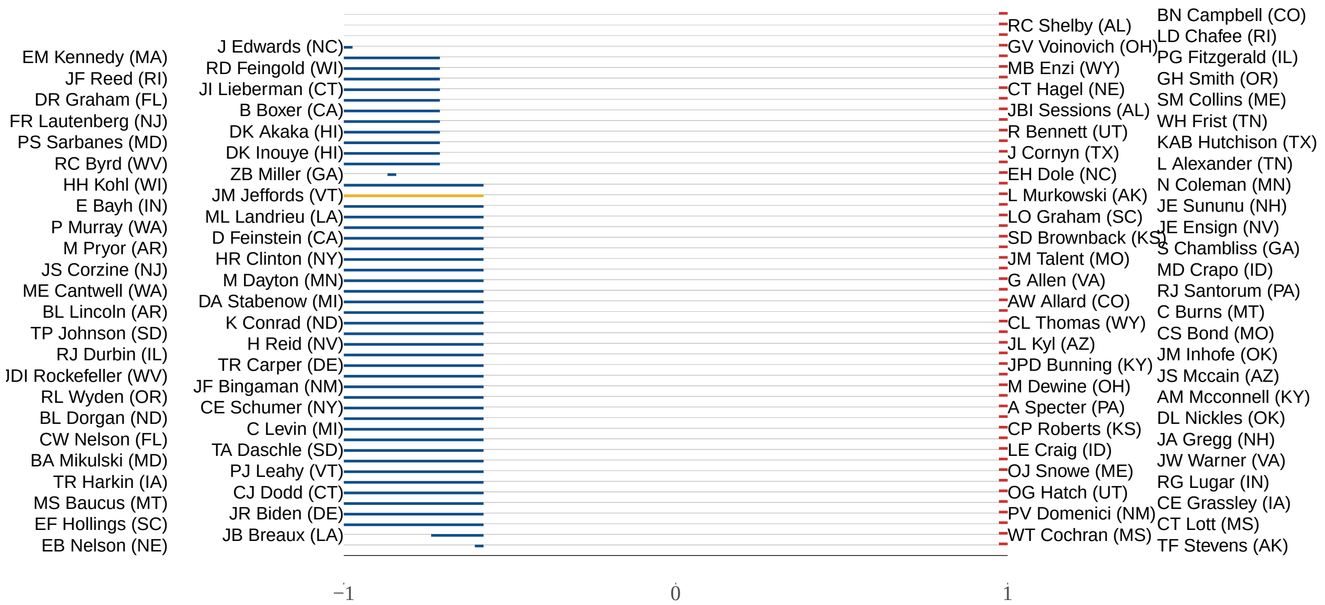

Figure 7. Interval representation of the 108th Senate.

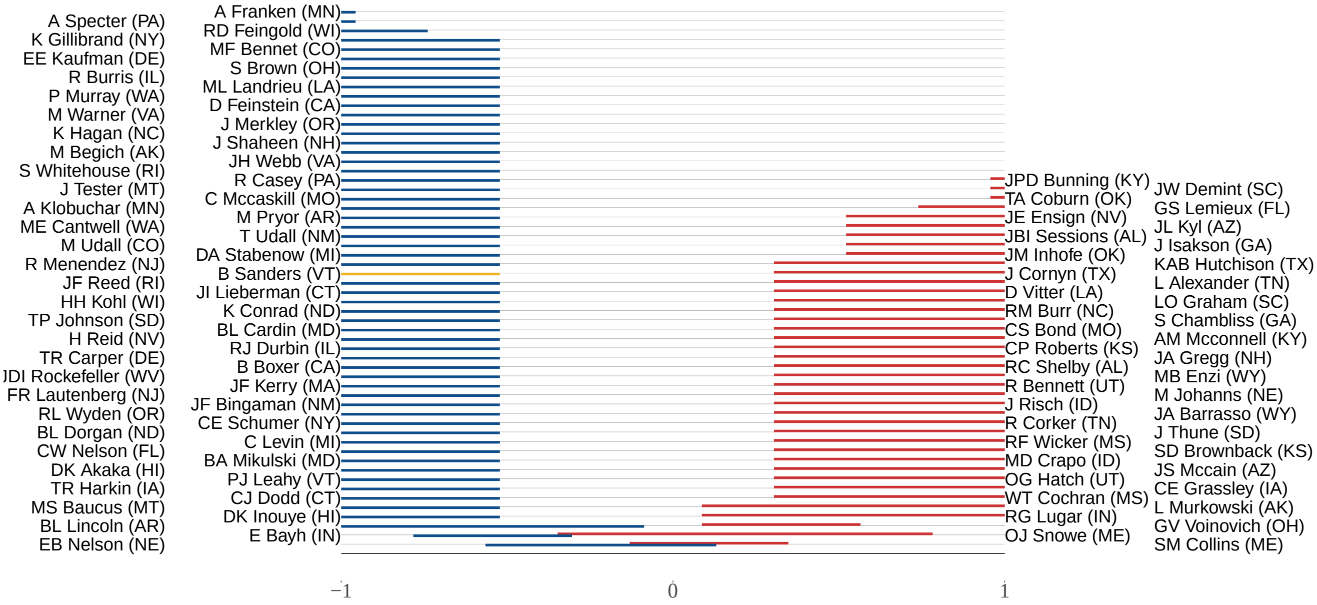

Figure 8. Interval representation of the 111th Senate.

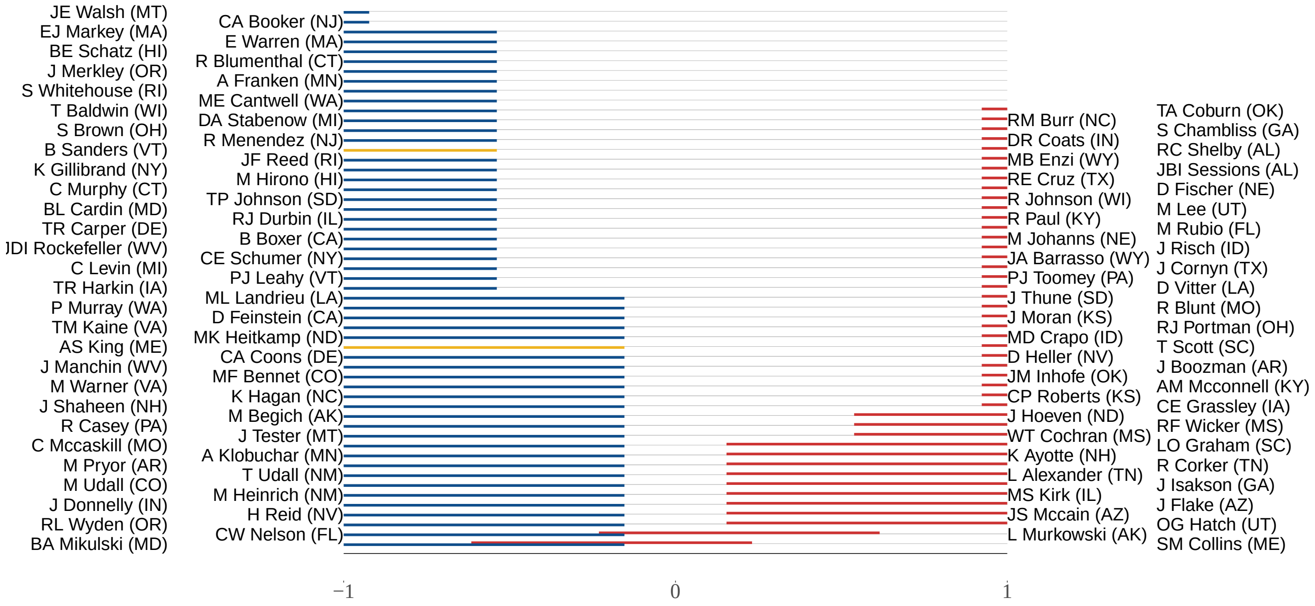

Figure 9. Interval representation of the 113th Senate.

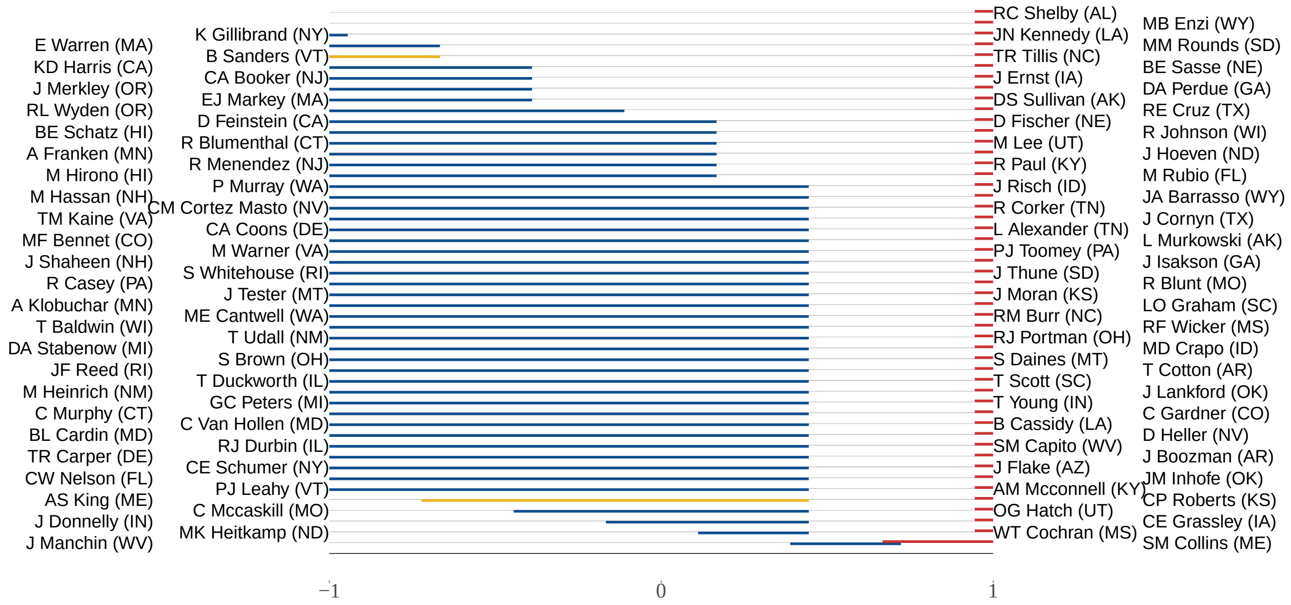

Figure 10. Interval representation of the 115th Senate.

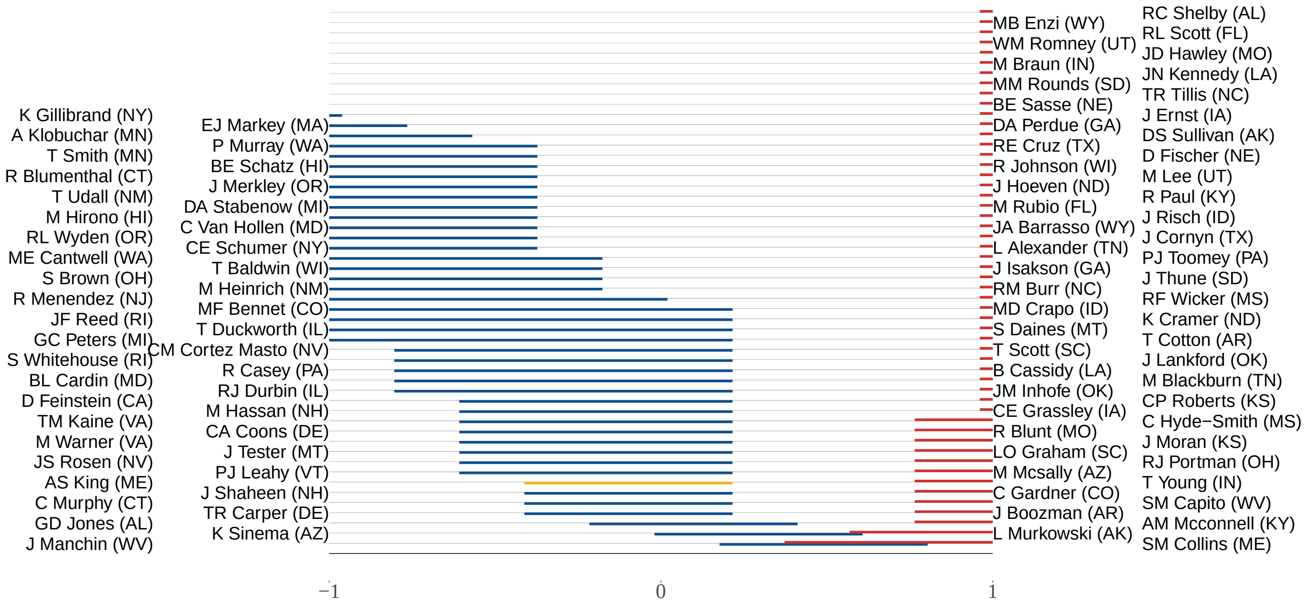

Figure 11. Interval representation of the 116th Senate.

Boxicity 2 representations

The figures below show the two dimensional boxes for the five niche overlap networks that were found to be boxicity 2 graphs. The figures are not annotated and structural equivalence classes are contracted (indicated by thickness in the interval representation).

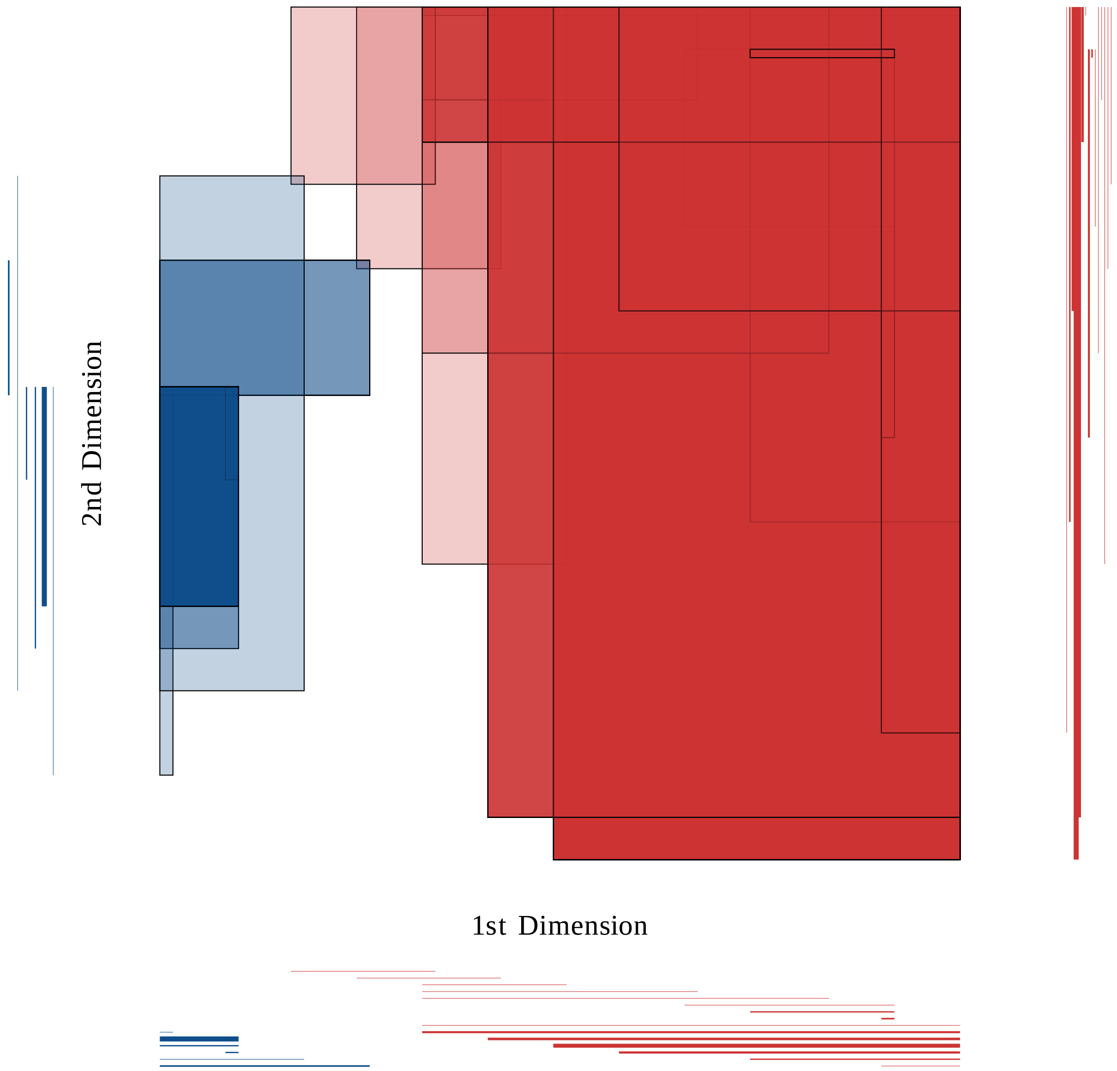

Figure 12. Box representation of the 105th Senate.

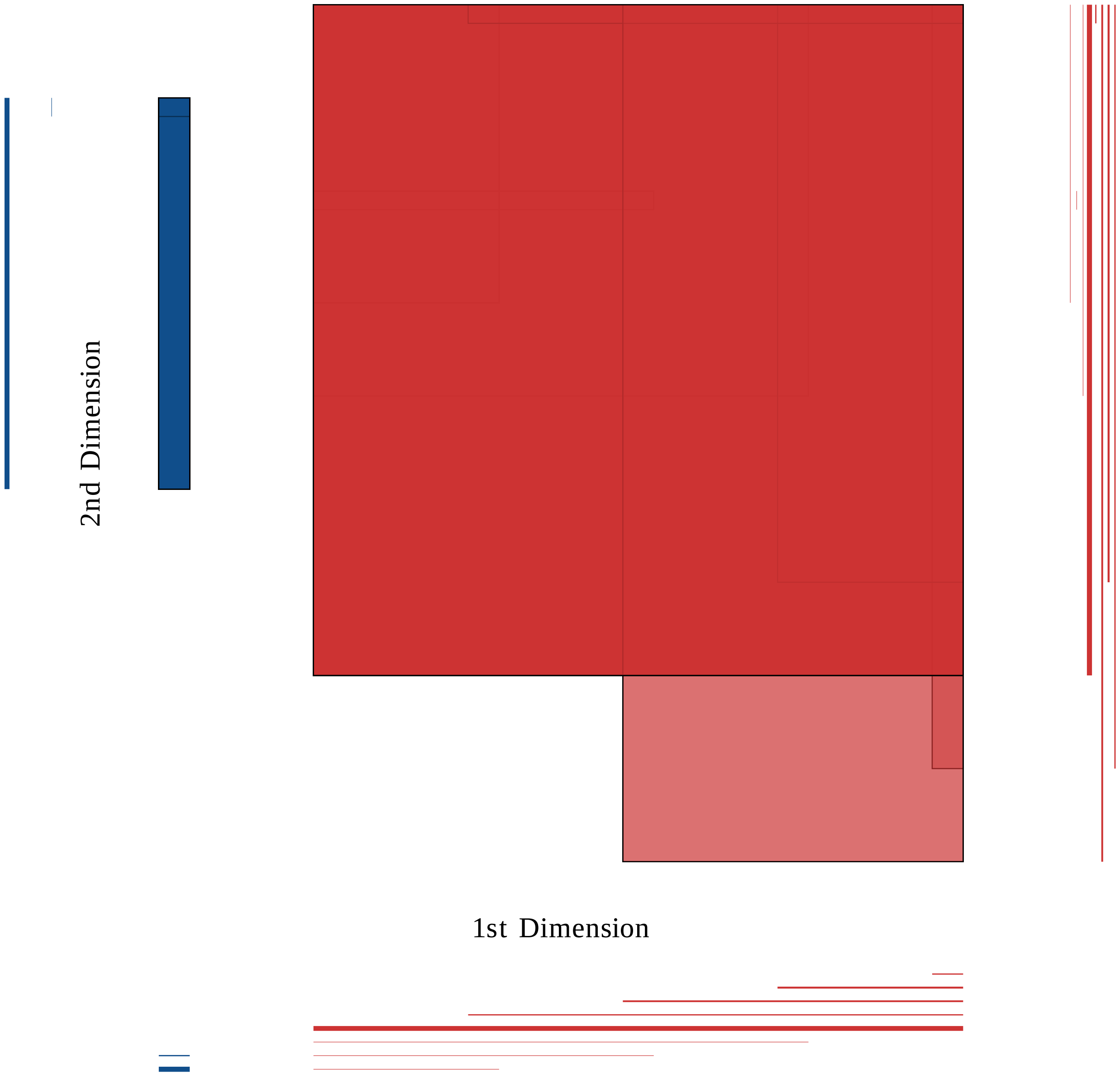

Figure 13. Box representation of the 106th Senate.

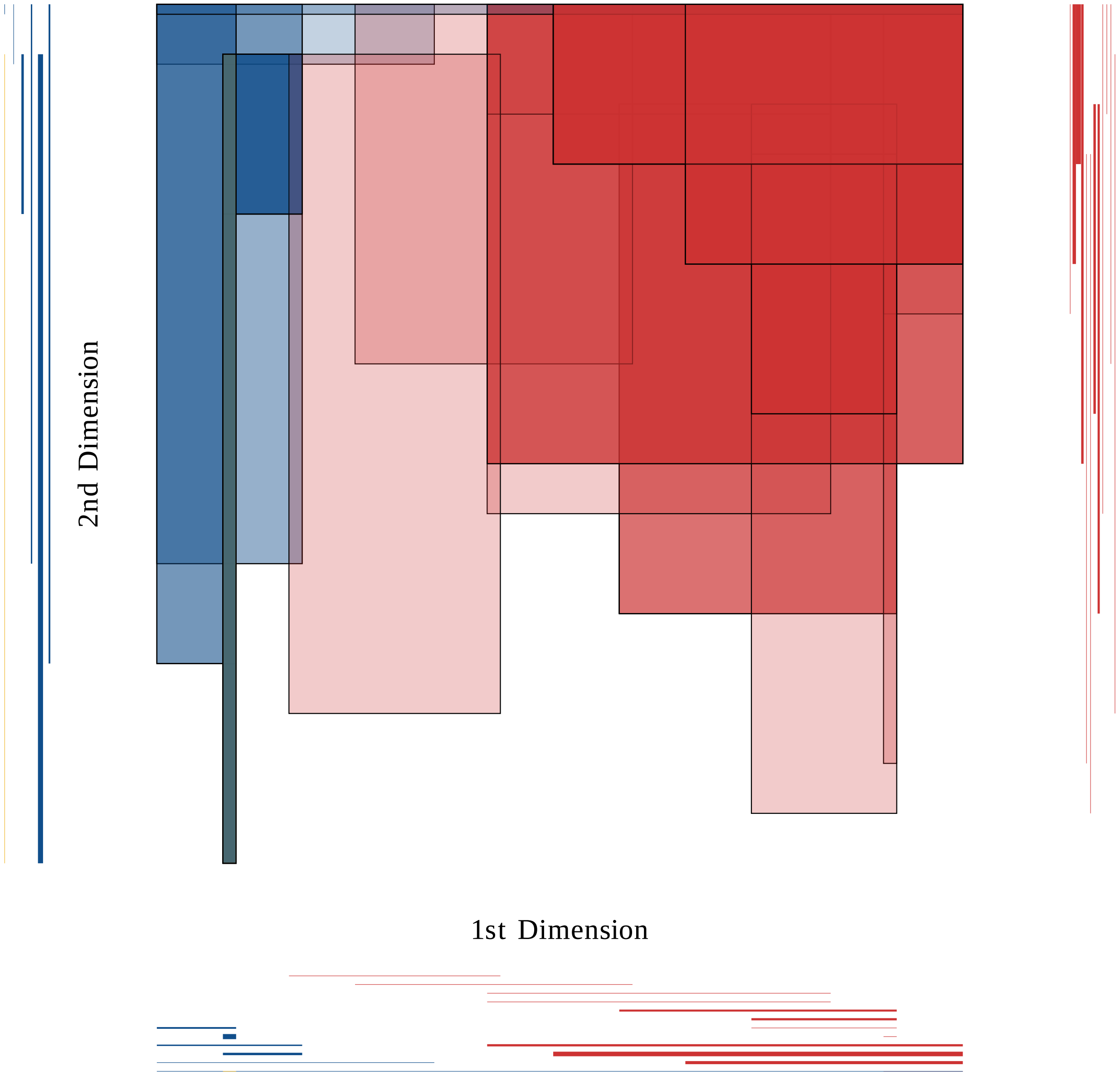

Figure 14. Box representation of the 109th Senate.

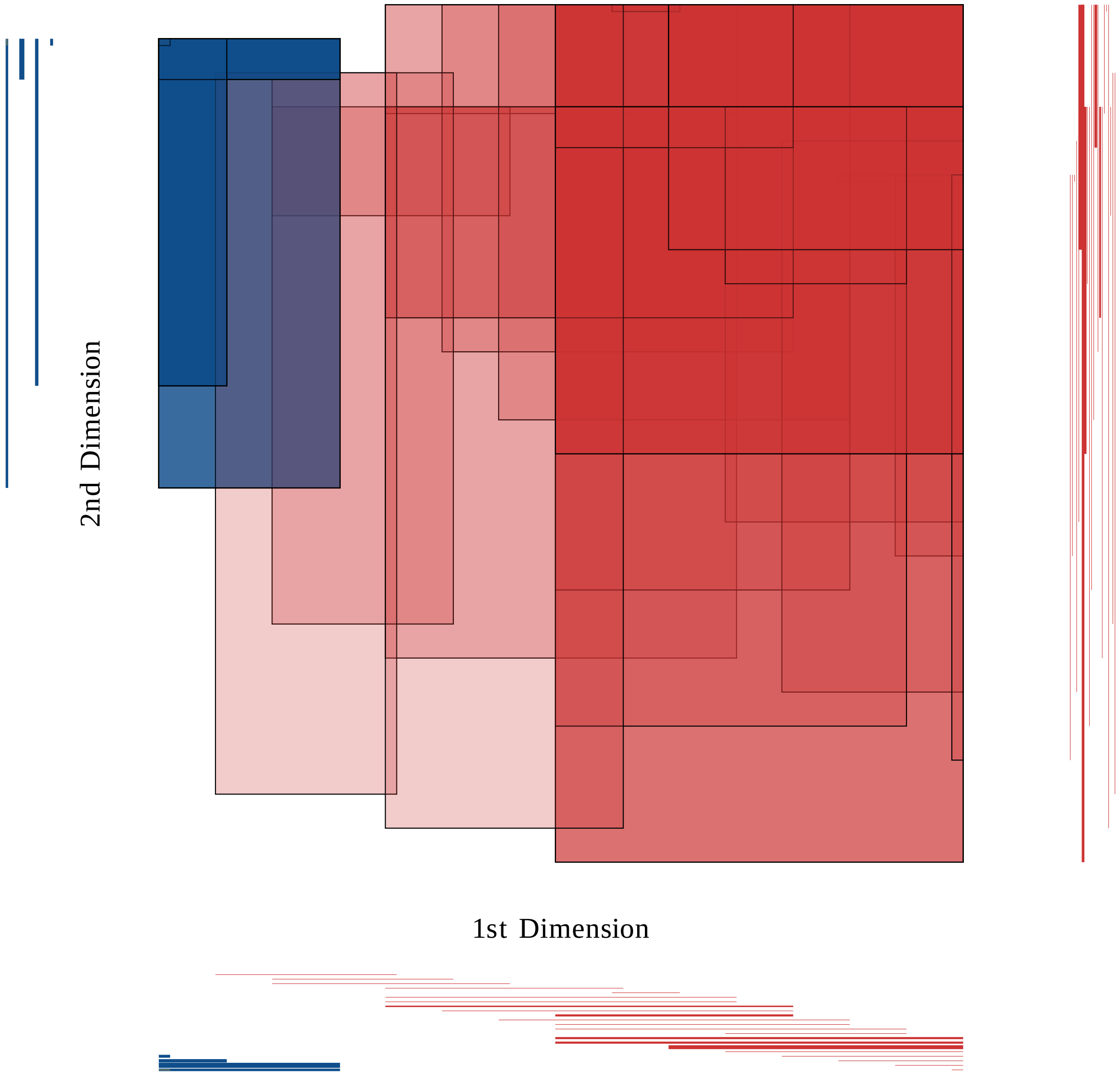

Figure 15. Box representation of the 112th Senate.

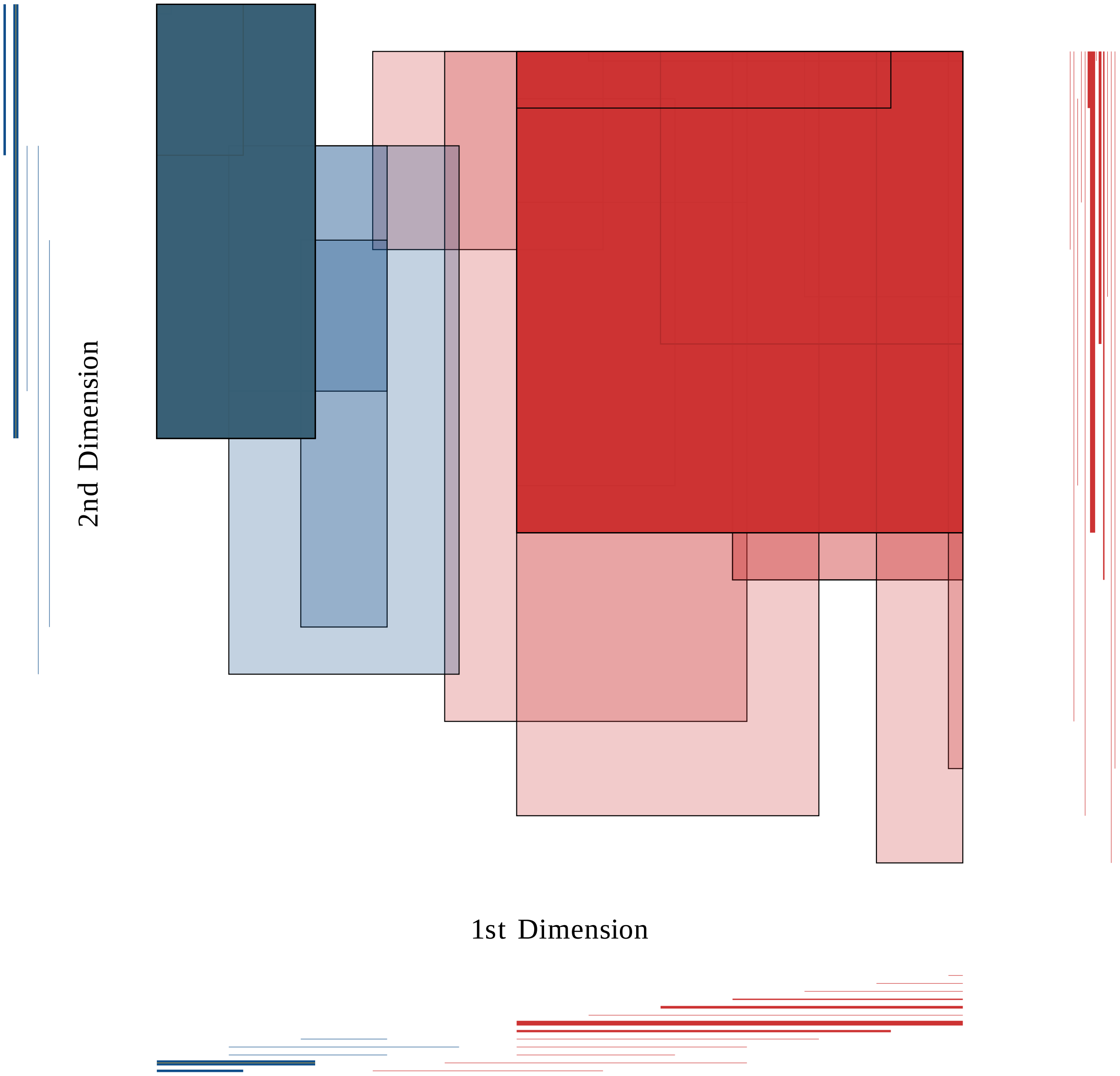

Figure 16. Box representation of the 114th Senate.

Niche point projections

Figure 17 shows the one dimensional niche point projections based on the Fiedler vector approach. These representations are used in the main paper to compute the distance between parties to assess polarization.

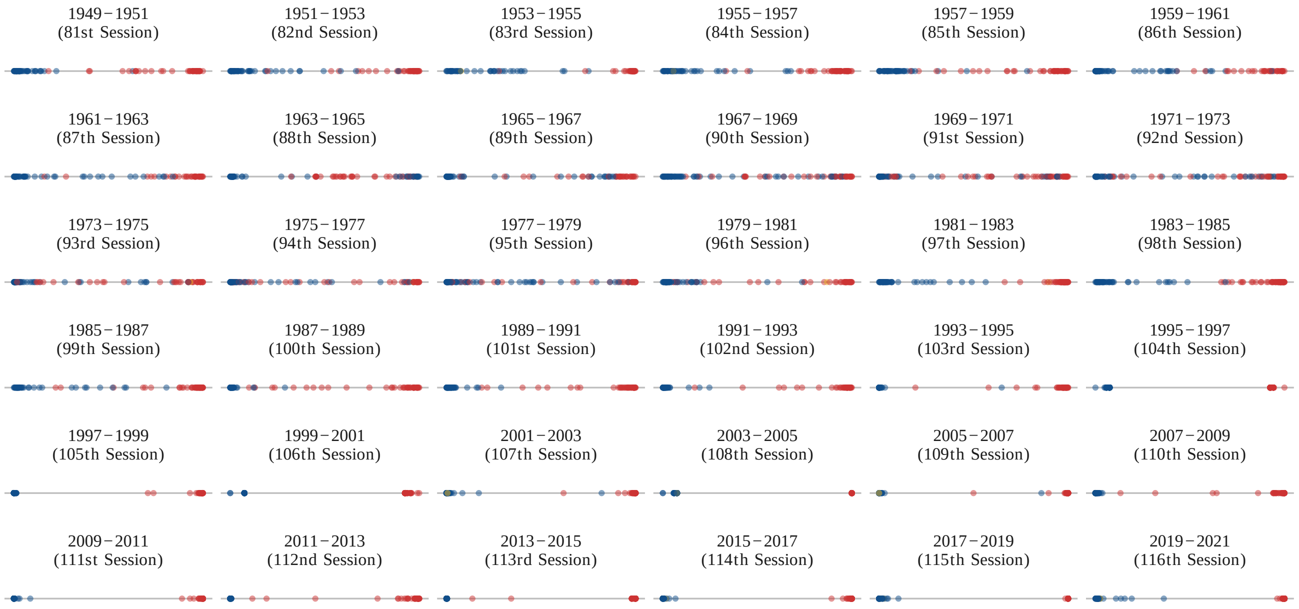

Figure 17. One dimensional niche point projections based on the Fiedler vectors.

Co-sponsorship networks

In this section, we repeat analyses of the main paper for bill co-sponsorship data (93rd to 116th Senate). The data was obtained from pro publica (`https://www.propublica.org`). The niche overlap networks obtained with the SDSM are shown in Figure 18. Figure 19 shows the normalized Lazarus count for the niche overlap networks of the co-sponsorship networks in comparison with the co-voting networks. None of the networks was found to be one dimensional. Although we do observe a general tendency toward a declining number of dimensions, it remains significantly higher than for the networks derived from roll-call votes. These results seem to weaken the observations from the main paper, however, they do confirm previous research where it was reported that the underlying space of bill co-sponsorship is of a higher dimensionality than for roll-call votes[13].

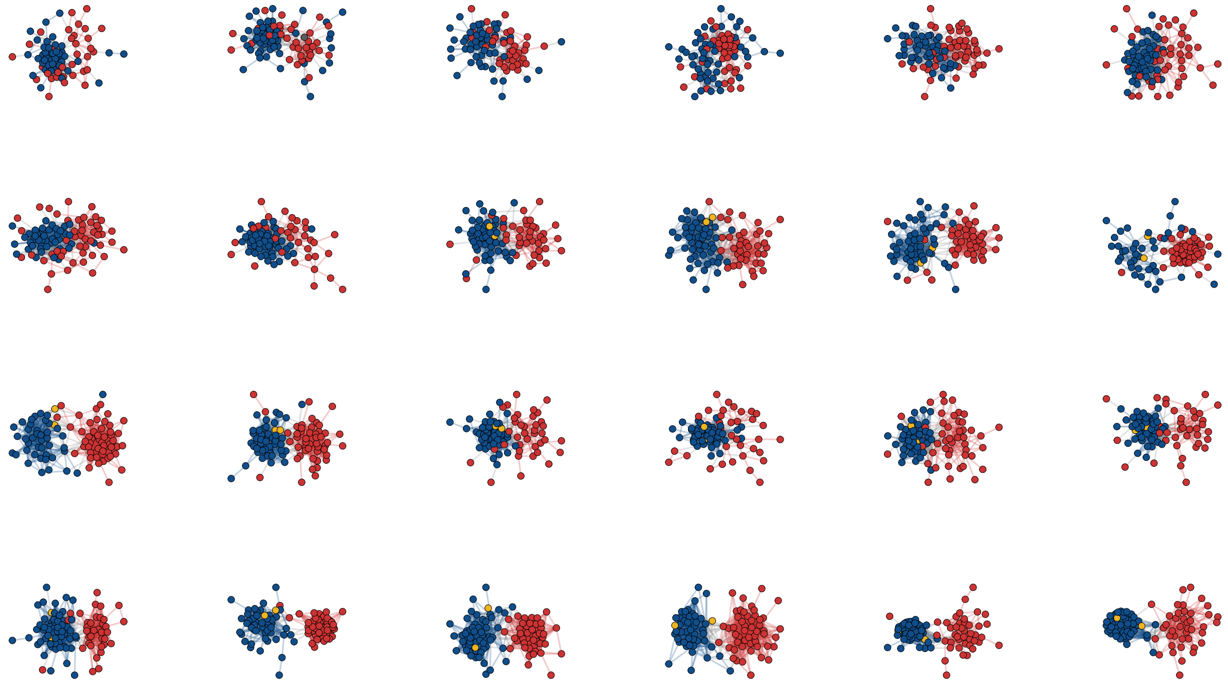

Figure 18. Niche overlap networks of senators inferred from bill co-sponsorships with the SDSM for the 93rd (top-left) to 116th Senate (bottom-right).

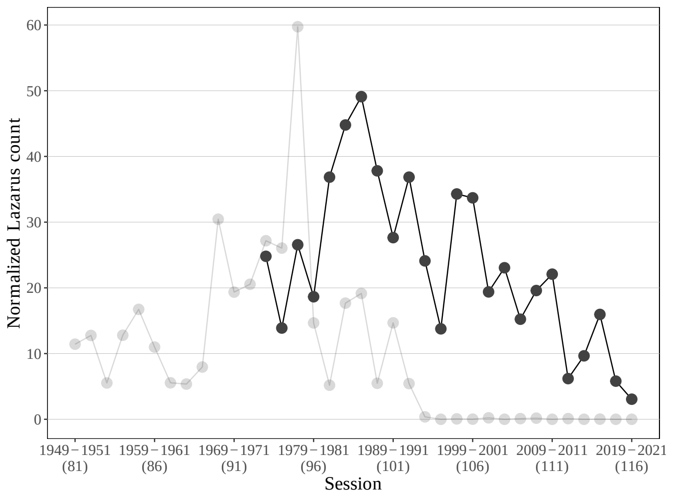

Figure 19. Upper bound for the normalized Lazarus counts obtained via simulated annealing of the niche overlap networks shown in Figure 18. Lazarus count of networks inferred from co-voting are shown in light grey.

References

1. R Core Team. *R: A Language and Environment for Statistical Computing*. R Foundation for Statistical Computing, Vienna, Austria (2020).
2. Fulkerson, D. & Gross, O. Incidence matrices and interval graphs. *Pac. journal mathematics* **15**, 835–855 (1965).
3. Atkins, J., Boman, E. & Hendrickson, B. A Spectral Algorithm for Seriation and the Consecutive Ones Problem. *SIAM J. on Comput.* **28**, 297–310, DOI: 10.1137/S0097539795285771 (1998).
4. Kendall, D. G. Incidence matrices, interval graphs and seriation in archeology. *Pac. J. mathematics* **28**, 565–570 (1969).
5. Quest, M. & Wegner, G. Characterization of the graphs with boxicity $\leqslant 2$. *Discret. Math.* **81**, 187–192, DOI: 10.1016/0012-365X(90)90151-7 (1990).
6. Domagalski, R., Neal, Z. & Sagan, B. Backbone: An R Package for Backbone Extraction of Weighted Graphs. *arXiv:1912.12779 [cs]* (2019). 1912.12779.
7. Garey, M. R. & Johnson, D. S. *Computers and Intractability*, vol. 29 (wh freeman New York, 2002).
8. Goldberg, P. W., Golumbic, M. C., Kaplan, H. & Shamir, R. Four Strikes Against Physical Mapping of DNA. *J. Comput. Biol.* **2**, 139–152, DOI: 10.1089/cmb.1995.2.139 (1995).
9. Golumbic, M. C. & Shamir, R. Complexity and Algorithms for Reasoning About Time: A Graph-theoretic Approach. *J. ACM* **40**, 1108–1133, DOI: 10.1145/174147.169675 (1993).
10. Chandran, L. S., Francis, M. C. & Sivadasan, N. Geometric Representation of Graphs in Low Dimension Using Axis Parallel Boxes. *Algorithmica* **56**, 129, DOI: 10.1007/s00453-008-9163-5 (2008).
11. Vuokko, N. Consecutive ones property and spectral ordering. In *Proceedings of the 2010 SIAM International Conference on Data Mining*, Proceedings, 350–360, DOI: 10.1137/1.9781611972801.31 (Society for Industrial and Applied Mathematics, 2010).
12. Applegate, D., Bixby, R., Chvatal, V. & Cook, W. Concorde TSP solver. *http://www.math.uwaterloo.ca/tsp/concorde/index.html* (2006).
13. Talbert, J. C. & Potoski, M. Setting the Legislative Agenda: The Dimensional Structure of Bill Cosponsoring and Floor Voting. *J. Politics* **64**, 864–891, DOI: 10.1111/0022-3816.00150 (2002).
